# Chromosomal phylogeny of Vampyressine bats (Chiroptera, Phyllostomidae) with description of two new sex chromosome systems

**DOI:** 10.1186/s12862-016-0689-x

**Published:** 2016-06-04

**Authors:** Anderson José Baia Gomes, Cleusa Yoshiko Nagamachi, Luis Reginaldo Ribeiro Rodrigues, Thayse Cristine Melo Benathar, Talita Fernanda Augusto Ribas, Patricia Caroline Mary O’Brien, Fengtang Yang, Malcolm Andrew Ferguson-Smith, Julio Cesar Pieczarka

**Affiliations:** Laboratório de Citogenética, CEABIO, ICB, Universidade Federal do Pará, Belém, Brazil; Laboratório de Genética e Biodiversidade, ICED, Universidade Federal do Oeste do Pará, Santarém, Brazil; Cambridge Resource Centre for Comparative Genomics, University of Cambridge Department of Veterinary Medicine, Cambridge, UK; Cytogenetics Facility, Welcome Trust Sanger Institute, Hinxton, South Cambridgeshire UK

**Keywords:** Chromosome phylogeny, Chromosome painting, Subtribe Vampyressina, Compound sex chromosome system, Sex determination

## Abstract

**Background:**

The subtribe Vampyressina (*sensu* Baker et al. 2003) encompasses approximately 43 species and seven genera and is a recent and diversified group of New World leaf-nosed bats specialized in fruit eating. The systematics of this group continues to be debated mainly because of the lack of congruence between topologies generated by molecular and morphological data. We analyzed seven species of all genera of vampyressine bats by multidirectional chromosome painting, using whole-chromosome-painting probes from *Carollia brevicauda* and *Phyllostomus hastatus*. Phylogenetic analyses were performed using shared discrete chromosomal segments as characters and the Phylogenetic Analysis Using Parsimony (PAUP) software package, using Desmodontinae as outgroup. We also used the Tree Analysis Using New Technology (TNT) software.

**Results:**

The result showed a well-supported phylogeny congruent with molecular topologies regarding the sister taxa relationship of *Vampyressa* and *Mesophylla* genera, as well as the close relationship between the genus *Chiroderma* and *Vampyriscus*.

**Conclusions:**

Our results supported the hypothesis that all genera of this subtribe have compound sex chromosome systems that originated from an X-autosome translocation, an ancestral condition observed in the Stenodermatinae. Additional rearrangements occurred independently in the genus *Vampyressa* and *Mesophylla* yielding the X1X1X2X2/X1X2Y sex chromosome system. This work presents additional data supporting the hypothesis based on molecular studies regarding the polyphyly of the genus *Vampyressa* and its sister relationship to *Mesophylla*.

**Electronic supplementary material:**

The online version of this article (doi:10.1186/s12862-016-0689-x) contains supplementary material, which is available to authorized users.

## Background

The subtribe Vampyressina (*sensu* Baker et al. 2003 [1]) corresponds to a diversified group of New World leaf-nosed bats specialized in fruit eating. This group encompasses 43 species of phyllostomid genera *Platyrrhinus*, *Vampyrodes*, *Uroderma*, *Chiroderma*, *Vampyriscus*, *Vampyressa* and *Mesophylla* [2–6]. Intergeneric relationships have been the focus of great debate, with disagreements mainly due to discrepant topologies generated by morphological and molecular data regarding the generic status of *Vampyressa* and *Vampyriscus* and their relationships with *Chiroderma*, *Ectophylla* and *Mesophylla* [1, 7–9].

Most studies based on morphological data support the sister taxa relationship between the genera *Ectophylla* and *Mesophylla* [7, 10, 11]. However, a study based on cranio-dental characters agrees with the molecular consensus about the close affinity of the genera *Mesophylla* and *Vampyressa* [12]. On the other hand, a study of restriction site variation of mitochondrial genes ND3 and ND4 supports the morphological view [13]. A reanalysis based on a direct survey on DNA sequences of these genes agrees with the molecular hypothesis [9].

Cytogenetic studies are agreed on the close relationship between *Mesophylla* and *Vampyressa*. All were based on gross chromosome morphology and the shared sex chromosome systems [14–17]. However, no cladistics analysis was made taking into account the different karyotypes of the species within this group.

Chromosome rearrangements are rare genomic changes (*sensu* [18]) and, because of their Mendelian inheritance, can be used for phylogenetic inferences. Phylogenetic studies based on chromosome data have contributed to the systematics of many groups of vertebrates, especially in Chiroptera [19–26].

In this paper, we use multidirectional chromosome painting with whole chromosome probes from two phyllostomid bats (*Carollia brevicauda* and *Phyllostomus hastatus*), as well as chromosome banding and Fluorescence *In Situ* Hybridization (FISH) with 18S rDNA and telomeric probes, to establish a genome-wide comparative chromosomal map for all genera of Vampyressine bats. Using chromosomal rearrangements as characters, we built a phylogeny that sheds some new light on the evolutionary relationships among these bats.

## Methods

### Specimens examined

Representative species of the subtribe Vampyressina were collected in natural habitats during field expeditions to different places in the Amazon Basin (Table 1). Specimens were maintained in the laboratory with food and water, free from stress, until their necessary euthanasia. This study was specifically approved by the Animal Ethics Committee (Comitê de Ética Animal) from Universidade Federal do Pará (Permit 68–2015) over and above the use of approved general protocols. *Voucher* specimens were fixed in 10 % formalin, preserved in 70 % ethanol, and deposited in the mammalian collections of the Museu Paraense Emilio Goeldi, Museu de Zoologia da Universidade Federal do Oeste do Pará and Coleção Zoológica do Instituto de Pesquisas do Amapá. JCP has a permanent field permit, number 13,248 from “Instituto Chico Mendes de Conservação da Biodiversidade”. The Cytogenetics Laboratory from UFPa has a special permit number 19/2003 from the Ministry of Environment for samples transport and 52/2003 for using the samples for research.

**Table 1:** Species analyzed in this work and from Pieczarka et al. (2005, 2013) and Sotero-Caio et al. (2011)

Species	Locality	Sample	2n	FN	Reference
*Platyrrhinus incarum*, PIN	Belém (1° 27’ 08” S; 48° 29’ 28” W)	1 F	30	56	This study
*Vampyrodes caraccioli*, VCA	Belém (1° 27’ 08” S; 48° 29’ 28” W)	2 M	30	56	This study
*Chiroderma villosum*, CVI	Juruti (2° 09’ 18” S; 56° 05’ 50” W) and Santarem (2° 26’ 57” S; 54° 41’ 59” W)	1 M, 1 F	26	48	This study
*Mesophylla macconnelli*, MMA	Faro (1° 13’ 01” S; 57° 44’ 03” W) and Cotriguaçu (9° 52’ 10” S; 58° 33’ 18” W)	2 M, 1 F	21/22	18	This study
*Vampyressa thyone*, VTH	Campos Novos (2° 22’ 12” N; 61° 26’ 08” W)	1 M, 1 F	23/24	20	This study
*Vampyriscus bidens*, VBI	Belém (1° 27’ 08” S; 48° 29’ 28” W) and Faro (1° 13’ 01” S; 57° 44’ 03” W)	2 M, 1 F	26	48	This study
*Vampyriscus brocki*, VBR	Lourenço (2° 19’ 49” N; 51° 37’ 08” W)	1 M	24	44	This study
*Uroderma magnirostrum*, UMA	–	–	36	62	Pieczarka et al. (2013)
*Uroderma bilobatum*, UBI	–	–	42	50	Pieczarka et al. (2013)
*Artibeus obscurus*, AOB	–	–	30/31	56	Pieczarka et al. (2013)
*Phyllostomus hastatus*, PHA	–	–	32	58	Pieczarka et al. (2005)
*Carollia brevicauda*, CBR	–	–	20/21	36	Pieczarka et al. (2005)
*Diphylla ecaudata*, DEC	–	–	32	60	Sotero-Caio et al. (2011)
*Diaemus youngi*, DYO	–	–	32	60	Sotero-Caio et al. (2011)
*Desmodus rotundus*, DRO	–	–	28	52	Sotero-Caio et al. (2011)

### Chromosomal preparation and chromosome banding

Metaphase spreads were obtained from bone marrow preparations after colchicine treatment, following [27] and [28], as well as from fibroblasts cultured according to [29]. G-banding was performed using trypsin treatment [30] and subsequent incubation in saline solution (0.5 × SSC) at 60 °C, and staining with Wright’s solution [31]. C-banding was carried out according to [32] and Ag-NOR staining followed [33].

### Fluorescence in Situ Hybridization (FISH)

FISH using telomeric probes (All Human Telomere Probes, Oncor) and 18S rDNA probes from *Prochilodus argenteus* [34] labeled with biotin or digoxigenin by nick translation was performed. Whole-chromosome-specific painting probes from *Phyllostomus hastatus* (PHA) and *Carollia brevicauda* (CBR) were obtained from flow-sorted chromosomes [25], labeled by DOP-PCR (Degenerate Oligonucleotide-Primed-Polymerase Chain Reaction) amplification [35] and hybridized as previously described [25, 36]. After hybridization and washing, metaphases were stained with DAPI (4’,6-diamidino-2-phenylindole), and images captured using the Axiovision 3.0 software (Carl Zeiss) through an AxioCam MRm coupled to a Zeiss-Axiophot 2 microscope.

### Data from literature

In previous publications [25, 37] the species *Phyllostomus hastatus*, *Carollia brevicauda, Artibeus obscurus*, *Uroderma bilobatum* and *U. magnirostrum* were analyzed using the same whole-chromosome probes as used here. We added those data to the present work, and included them in our phylogenetic analysis. Sotero-Caio et al. [38] analyzed *Diphylla ecaudata*, *Diaemus youngi* and *Desmodus rotundus* also using the same probes. These species (Desmondontinae) were used as outgroup on phylogenetic analysis because of their basal position in published molecular phylogenies and because this is a monophyletic subfamily with well supported molecular [1] and chromosomal [38] synapomorphies.

### Phylogenetic analysis

A data matrix was established where the chromosomal rearrangements are the characters under analysis, and discrete chromosomal syntenic blocks are the characters states that reflect the occurrence of rearrangements (Additional file 1: Table S1). The data matrix also included species previously analyzed [25, 37, 38], and it is available as a supplementary file. Maximum parsimony (MP) analysis was made using PAUP 4.0b10 [39]. A heuristic search to find most parsimonious tree(s) was performed using Tree Bisection Reconnection (TBR) branch-swapping; the bootstrap posterior probability was obtained with one thousand replicates. The Bremer support or “decay index” [40, 41] was calculated to verify the inconsistency of the branches in the consensus tree using the software “Tree Analysis Using New Technology” (TNT) version 1.1 [42], freely distributed by the Willi Hennig Society.

## Results

### Karyotype description and multidirectional chromosome painting in Chiroderma villosum (CVI)

**Fig. 1:**
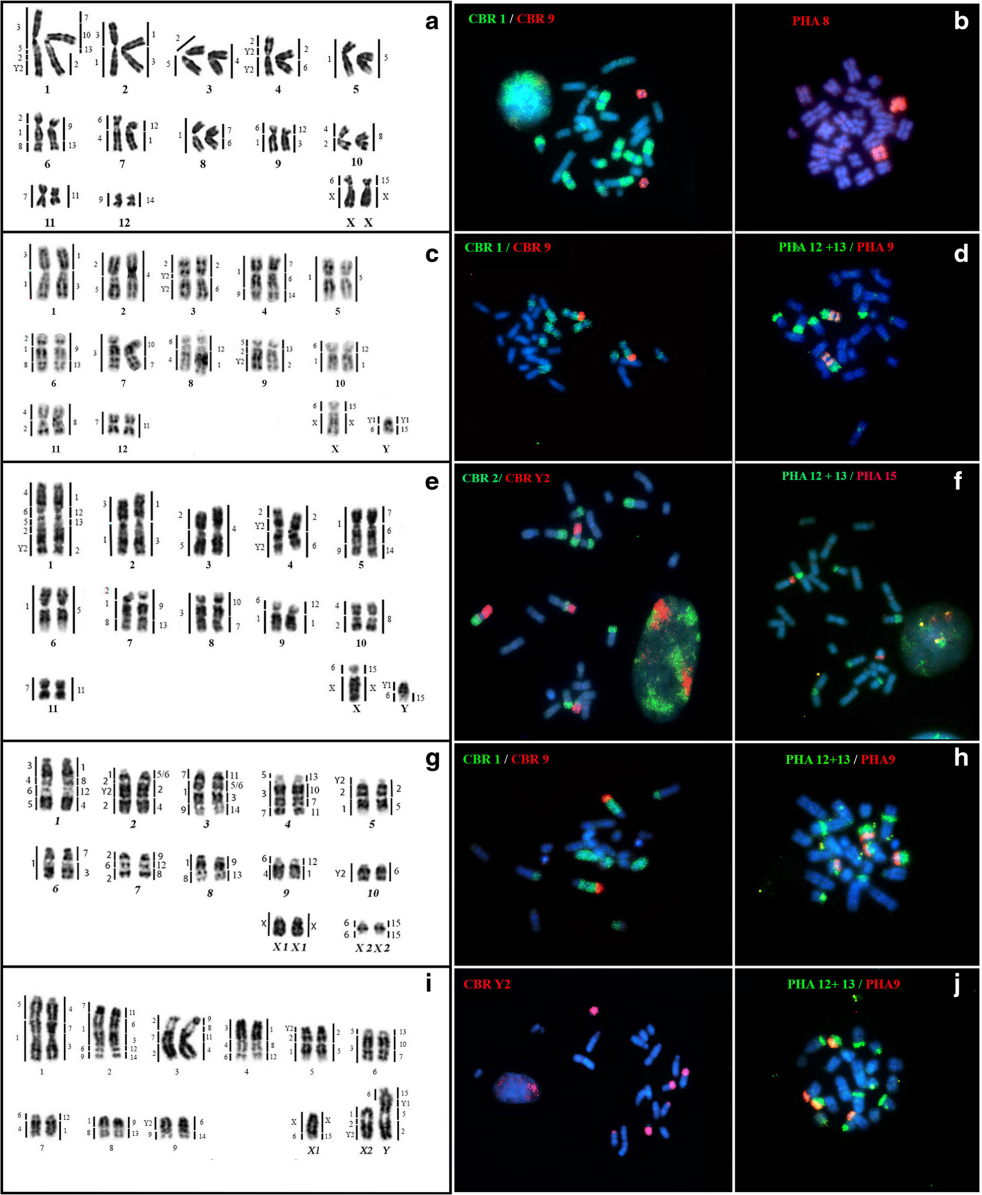
Left: G-banding with mapping of *Carollia brevicauda* (left) and *Phyllostomus hastatus* (right) probes. Right: Examples of chromosome painting on the analyzed species with *Carollia brevicauda* (CBR) and *Phyllostomus hastatus* (PHA) probes. The probes were labeled with biotin; the green probe was detected with FITC; the red one with Cy3. DAPI was used as counterstaining. Each line represents one species. **a** and **b**: *Chiroderma villosum*; **c** and **d**: *Vampyriscus bidens*; **e** and **f**: *Vampyriscus brocki*; **g** and **h**: *Vampyressa thyone*; **i** and **j**: *Mesophylla macconnelli*

We analyzed two specimens of *C. villosum* (Table 1). Both samples show 2n = 26 chromosomes and Fundamental Number, FN = 48 (Fig. 1, CVI). The autosome complement consists solely of biarmed elements (meta, submeta and subtelocentric). The X is a medium-sized subtelocentric and the Y is a small acrocentric chromosome. The constitutive heterochromatin (CH) is restricted to centromeric regions of all chromosomes, including the X (Additional file 2: Figure S1, CVI). FISH using telomeric probes show signals at the distal ends of chromosomes, with dominant strong signals at the centromeric regions of all but pairs 6, 8, 11 and the Y (Additional File 3: Figure S2, CVI). Silver staining (not shown) and FISH with 18S rDNA revealed a single Nucleolar Organizer Region (NOR) in the short arm of pair 7 (Additional File 3: Figure S2, CVI).

Multidirectional chromosome painting using whole-chromosome probes from *Carollia brevicauda* (CBR) and *Phyllostomus hastatus* (PHA) revealed 25 and 23 shared conserved syntenic segments, respectively, with the number of syntenic blocks ranging from one to four on each chromosome of CVI (Fig. 1, CVI).

### Karyotype description and multidirectional chromosome painting in Vampyriscus bidens (VBI)

All five specimens of *V. bidens* (Table 1) collected at different sites in the Brazilian Amazon have a 2n = 26 and FN = 48, with all chromosomes biarmed. The X is subtelocentric and the Y is acrocentric (Fig. 1, VBI). The CH is located at the centromeric region of chromosomes and in the proximal region of the Y chromosome (Additional file 2: Figure S1, VBI). Hybridization with telomeric probes showed signals at the tips of chromosomes and weaker signals at the centromeric regions of some chromosomes in association with the CH (Additional file 3: Figure S2, VBI). FISH using 18S rDNA probes, and silver staining, showed two NOR located on pairs 8 and 9 (Additional file 3: Figure S2, VBI).

Chromosome painting using CBR and PHA probes revealed 25 and 23 conserved segments on *V. bidens*, respectively. The number of syntenic blocks ranges from one to three in different VBI chromosomes (Fig. 1, VBI).

### Karyotype description and multidirectional chromosome painting in Vampyriscus brocki (VBR)

*Vampyriscus brocki* collected in Amapá State has a 2n = 24 and a FN = 44 (Fig. 1, VBR). Its karyotype, as in the congeneric VBI, has only biarmed elements (meta, submeta and subtelocentric). The X is subtelocentric and the Y is acrocentric. The CH is located in the pericentromeric regions of chromosomes and the proximal part of Y (Additional file 2: Figure S1, VBR). FISH signals with telomeric probes were detected at the tips of chromosomes (Additional file 3: Figure S2, VBR). Signals from an 18S rDNA probe were detected at the distal portion of the short arm of pairs 5, 7, 8, and 9; in agreement with silver staining results (Additional file 3: Figure S2, VBR).

Chromosome painting using CBR and PHA probes detect 25 and 23 syntenic segments, respectively. We found a maximum of five segments per chromosome with CBR probes and four with PHA (Fig. 1, VBR).

### Karyotype description and multidirectional chromosome painting in Vampyressa thyone (VTH)

Two specimens of *V. thyone* caught from Serra of Apeú in Roraima state were karyotyped and show a 2n = 24 complement in the female and 2n = 23 in the male, with FN = 20 (Fig. 1, VTH). The chromosome complement has only uniarmed pairs, and the sex chromosome system is X1X1X2X2/X1X2Y, so there is one chromosome less in the male karyotype. The CH is located in small bands in the pericentromeric regions of chromosomes, with one strong CH block in the interstitial part of ×2 (Additional file 2: Figure S1, VTH). FISH using the telomeric probe showed signals at the tips of chromosomes (Additional file 3: Figure S2, VTH); the 18S rDNA probe revealed two NORs on pairs 5 and 9 (Additional file 3: Figure S2, VTH), confirming previous results from silver nitrate (AgNOR) staining.

Chromosome painting revealed 28 and 29 shared segments with CBR and PHA probes, respectively. Results from each set of probes showed a maximum of four syntenic blocks per chromosome in VTH (Fig. 1, VTH).

### Karyotype description and multidirectional chromosome painting in Mesophylla macconnelli (MMA)

We analyzed three specimens of *M. macconnelli*. The diploid number is 2n = 22 in female and 2n = 21 in males, with the FN = 18 (Fig. 1, MMA). The chromosomal complement has only uniarmed pairs. The sex chromosome system is X1X1X2X2/X1X2Y. The CH is present on small bands in the pericentromeric region of the autosomes and on the proximal part of the Y (Additional file 2: Figure S1, MMA). Probes revealed telomeric signals on the distal ends of all chromosomes (Additional file 3: Figure S2, MMA). Staining with silver nitrate (Ag-NOR) and FISH with 18S rDNA probe showed one signal in the distal portion of pair 2 (Additional file 3: Figure S2, MMA).

Chromosome painting on MMA metaphases revealed 28 syntenic segments using probes from CBR, and 30 syntenic segments using probes from PHA. We found a maximum of four segments per chromosome matching CBR probes, and five matching PHA probes (Fig. 1, MMA).

### Karyotype description of Platyrrhinus incarum (PIN) and Vampyrodes caraccioli (VCA)

We analyzed single specimens of *P. incarum* and *V. caraccioli*. Both species have a 2n = 30, FN = 56 complement and a Neo-XY sex chromosome system (Additional file 4: Figure S3, PIN and VCA, respectively). The CH is located in the pericentromeric region of all chromosomes for PIN (Additional File 2: Figure S1, PIN) and in the centromeric regions of the chromosomal complement of VCA (Additional file 2: Figure S1, VCA); additional heterochromatic blocks are present at the distal ends of the short arms of chromosomes 5, 7 and 13 in both species. In PIN, FISH with telomeric probes produced signals at the tips of chromosomes and in the centromeric regions of pair 13 for both species. Additionally, minor signals in some pairs co-located with CH for PIN (Additional file 3: Figure S2, PIN). In VCA, telomeric probes detect signals at the tips of chromosomes and the centromeric regions of pairs 1, 5 and 13 (Additional file 3: Figure S2, VCA). Probe signals from 18S rDNA are located in pair 7, confirming the silver staining in PIN (Additional file 3: Figure S2, PIN), and in chromosome pairs 5 and 7 in VCA.

The banding patterns of these two species (PIN and VCA) are similar to those of *Artibeus obscurus* (Additional file 4: Figure S3 [37]. The only differences found are a pericentric inversion on pair 5 in PIN, the multiple sex chromosome system in *A. obscurus* (XX/XY1Y2) and the Neo-XY in PIN and VCA. Because of the similar G-banded karyotypes in PIN, VCA, and AOB, we infer that the syntenic groups would be the same as in AOB.

### Phylogenetic analyses using chromosome as characters

**Fig. 2:**
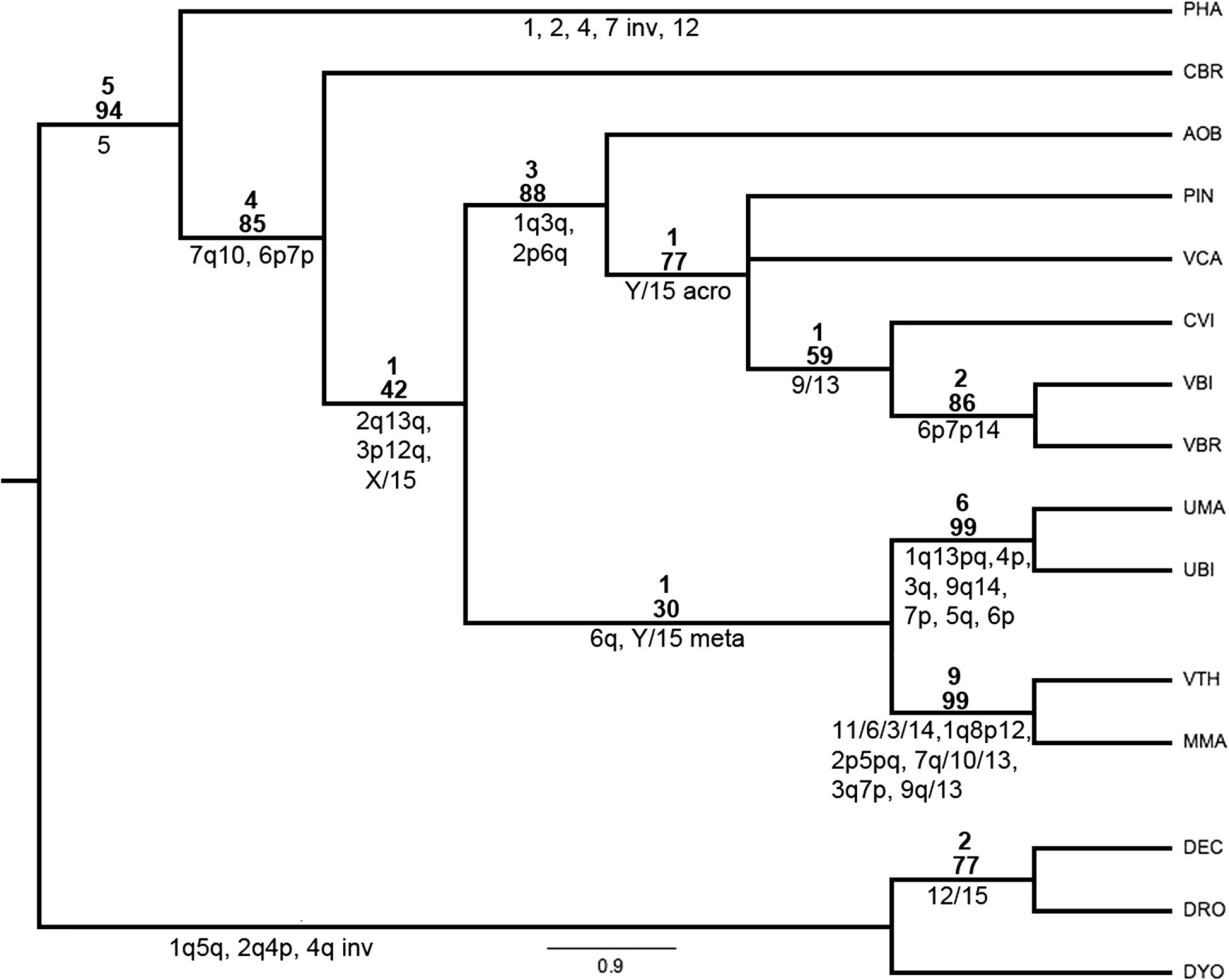
Maximum parsimony tree obtained after PAUP analysis of chromosomal characters found in representative species of Vampyressinae bats. Numbers above branch are Bremer decay index and bootstrap values for 1000 replicates and below are the shared syntenic blocks. The abbreviations of the name of species are detailed in Table 1. The characters “Y/15 acro” and “Y/15 meta” are different chromosomes and resulted from different rearrangements. While in the character 9/13 includes both these chromosomes in full, 9q/13 includes the 13 and the long arm of 9. Figure 3 details the evolution of the sex chromosome systems; note that it matches perfectly with the phylogeny shown here

We analyzed all representative genera in Vampyressina. Multidirectional chromosome painting identified chromosome homologies and we used a total of 86 discrete chromosomal characters to build a matrix of their presence or absence (Additional File 1 Table). The Maximum Parsimony analysis (MP) resulted in one most parsimonious tree (Tree length = 115, Consistence index = 0.7478, retention index = 0.7680, Homoplasy index = 0.2522). The main branch leads to all analyzed species except the outgroup (Bremer index 5, Bootstrap 95). After the split from PHA, the next branch (Bremer index 4, Bootstrap 85) leads to CBR (Carolliinae) and Vampyressina (Stenodermatinae). Then the Stenodermatinae branch (Bremer index 1, Bootstrap 42) splits in two branches. The first branch (Bremer index 3, Bootstrap 88) leads to AOB, PIN, VCA, CVI, VBR and VBI. AOB is the first species to split followed by a branch (Bremer index 1, Bootstrap 77) with a polytomy involving PIN, VCA and a branch leading to CVI (Bremer index 1, Bootstrap 59) followed by VBI and VBR (Bremer index 2, Bootstrap 86). The second branch (Bremer index 1, Bootstrap 30) consists of the genus *Uroderma* (Bremer index 6, Bootstrap 99)*,* a sister branch of *Mesophylla* and *Vampyressa* (Bremer index 9, Bootstrap 99; Fig. 2).

## Discussion

### Genera Platyrrhinus and Vampyrodes

There are only two recognized species in *Vampyrodes* [6], whereas *Platyrrhinus* has at least 21 currently described species [5, 43, 44]. Both genera have the same 2n = 30 and FN = 56 karyotype, as well as the Neo-XY sex chromosome system (found in all species described thus far [14, 15, 45]). G-banding patterns have been published only for *Platyrrhinus*.

In our phylogeny, *P. incarum* and *V. caracciolli* share a polytomy along with the branch corresponding to the genera *Vampyriscus* and *Chiroderma*. This polytomy of *P. incarum* and *V. caracciolli* can be explained by their similarity in G-banding pattern and presence of only some autapomorphies. Since these species have no synapomorphies, their precise positions on the phylogeny cannot be determined, despite molecular studies suggest a close relationship among *Vampyrodes, Platyrrhinus, Vampyriscus* and *Chiroderma* [9]. The chromosomal homeology among these species and *Artibeus obscurus* supports the hypothesis that the 2n = 30–31 karyotype is probably the ancestral condition for the Stenodermatinae [46]. We found only two characteristics supporting the close relationship of *Platyrrhinus* and *Vampyrodes*, the shared NOR location observed on pair 7 and an interstitial telomeric site found on pair 13. These characteristics were not included in our phylogeny due to the variability of these marks in the Stenodermatinae. Because of the retention of several ancestral characters and the NOR position, we conclude that the genera *Platyrrhinus* and *Vampyrodes* probably are sister taxa, in accordance with most studies based on either the morphological or molecular data [7, 9, 43, 47].

### Inter- and intraspecific variation in the genus Uroderma

*Uroderma* shows wide karyotypic variation when compared with other species in Stenodermatinae. Three chromosomal races have been described for *U. bilobatum* (Baker et al. 1972 [48], 1975 [49]). Specimens with 2n = 42 and FN = 50 occur in South America, east of the Andes (2n = 42 race). Individuals with 2n = 38 and FN = 44 are distributed on the Atlantic side of Guatemala, Honduras and Yucatan Peninsula of Mexico (Central America) and along the Pacific coast of Colombia and northern Ecuador (2n = 38 race). Specimens with 2n = 44 and FN = 48 occur in the Pacific coast of Central America in El Salvador, Guatemala, Honduras and México (2n = 44 race). A hybrid zone was found in Honduras, but introgression between the two races is low (Hoffmann et al. 2008 [46]). This hybrid zone has been extensively studied since the second half of the 20^th^ century and there is major controversy regarding primary and secondary origins of the zone, the role of selective force in its maintenance, and the amount of gene flow across this zone (Baker et al. 1972 [48], Hoffmann et al. 2008 [46]).

Our data support the hypothesis that the 2n = 42 race is distributed throughout South and Central America. Geographic barriers such as the Andean mountains and probably the Gulf of Fonseca (which was under water throughout much of the Pleistocene) separated the race with 42 chromosomes, and gave rise to the 44 and 38 cytotypes (Hoffmann et al. 2008 [46]). There is temporal agreement between the geographic barrier and divergence of the three races that happened around 0.9 to 0.2 Mya (Hoffmann et al. 2008 [46]). Baker et al. (1982 [50]) have proposed the rearrangements that took place to form the cytotypes with 42, 44 and 38 chromosomes. We suggest a different set of events: first a pericentric inversion and a fusion/fission differentiated the races with 42 and 44 chromosomes; second a translocation and a tandem fusion from a karyotype with 2n = 42 would explain the variation observed in races with 38 chromosomes. Thus, we agree with Hoffmann et al. (2008 [46]) that the ancestral chromosomal race on the deepest branch could have 42 chromosomes, and that the hybrid zone arose later.

### Intergeneric relationships among Vampyressina

Several studies, based mainly on morphological data, support *Vampyressa* as a monophyletic group (Goodwin 1963 [10], Owen 1987 [11], Wetterer et al. 2000 [7]). However, there is consensus from molecular data regarding its *Vampyriscus* and *Chiroderma*, indicating that *V. bidens*, *V. brocki* and *V. nymphaea* can be grouped instead in the genus *Vampyriscus* and that only *V. pusilla, V. melissa,* and *V. thyone* belong to the genus *Vampyressa* (Baker et al. 2000 [51], 2003 [1], Porter and Baker 2004 [8], Hoofer and Baker 2006 [9]).

Our results agree with the phylogenetic analysis of nuclear TSHB-I2 sequences made by Hoofer et al. (2008 [52]), strongly supporting the monophyly of *Vampyriscus bidens* with V. *brocki* (and their relationship with *Chiroderma*), the sister taxon relationship between *Mesophylla* and *Vampyressa*, as well as the polyphyly of *Vampyressa*. As pointed out by those authors, these results are in strong agreement with previous molecular studies by Baker et al. (2003 [1]).

Despite our results showing similar pairwise intergeneric relationships as the molecular studies, we found different branching between these groups. Although our analysis recovered a basal branching for the non-Stenodermatinae outgroups (PHA and CBR), and the monophyly of Stenodermatinae, the position of AOB within the Vampyressina disagrees with molecular and morphological data. Nevertheless, the presented topology is strongly supported by chromosomal data, namely the rearrangements shown in Fig. 2. For instance, both branches show an Y/15 translocation, but the resulting chromosomes have different morphologies, suggesting that they have different origins. Alternatively, they could have undergone further rearrangements since their divergence from their most recent common ancestor or be a case of hemiplasy (Robison et al. 2008 [53]) where the ancestral population could have contained both forms in polymorphic fashion, each cytotype becoming fixed in a different branch. This fusion is an easily recurrent rearrangement, since all the species in both branches have an X/15 fusion. During meiosis the Y and the free autosome (the other 15) will be very close because of pairing with the X/15, thus increasing the possibility that any chromatin break in the Y and free 15 would result in a fusion. Also, *Artibeus obscurus* has the X/15 fusion only, but not Y/15. This genus is divided into two groups, large and small *Artibeus*. For some authors the large should be organized in the subgenus *Artibeus* and small in *Dermanura* (Redondo et al. 2008 [54]; Wilson & Reader, 2005 [55]) while others consider that these groups are distinct genera (Baker et al. 2003 [1]; Solari et al. 2009 [56]). All karyotypes already described for large *Artibeus* have a XY1Y2 system, but there is variation in *Dermanura*: *toltecus* and *aztecus* have a XY1Y2 system (Baker, 1973 [57]) while *watsoni* and *phaeotis* have neo-XY (Baker, 1967 [58]; Hsu et al. 1968 [59]). *A. cinereus* has a XY1Y2 system in Central America (Baker, 1973 [57]) and neo-XY in South America (Souza & Araujo, 1990 [45]; Noronha et al. 2010 [60]). The neo-XY in *Dermanura* represents a third fusion between the Y and 15.

We tested many data matrices, using both PAUP and TNT, and all the resulting trees have the same branching pattern, suggesting that Vampyressina could be a polyphyletic subtribe resulting from convergent evolution, in a similar fashion to that observed in the subfamilies of nectarivorous bats (Ribeiro et al. 2003 [61]; Sotero-Caio et al. 2013 [62]). Although some branches have low bootstrap values, the decay index values give them a good support. Whereas the bootstrap is a more quantitative test, the Bremer index is qualitative, as is chromosomal variation.

### Sex chromosome evolution in the subtribe Vampyressina

Translocations between an autosome and a sex chromosome are rarely fixed in speciation because of meiotic problems, including inactivation of the homologues fused to the X chromosome (Mattei et al. 1982 [63]). Phyllostomidae is an exception to this rule where there are subfamilies with most or all species having multiple or compound sex chromosome systems (e.g., Stenodermatinae and Carolliinae - Hsu et al. 1968 [59], Tuker 1986 [64]). In this work, we present for the first time a hypothesis explaining the sex chromosome evolution in the Vampyressina and describe the events that gave rise to the sex chromosome systems observed in *M. macconnelli* and *V. thyone*.

**Fig. 3:**
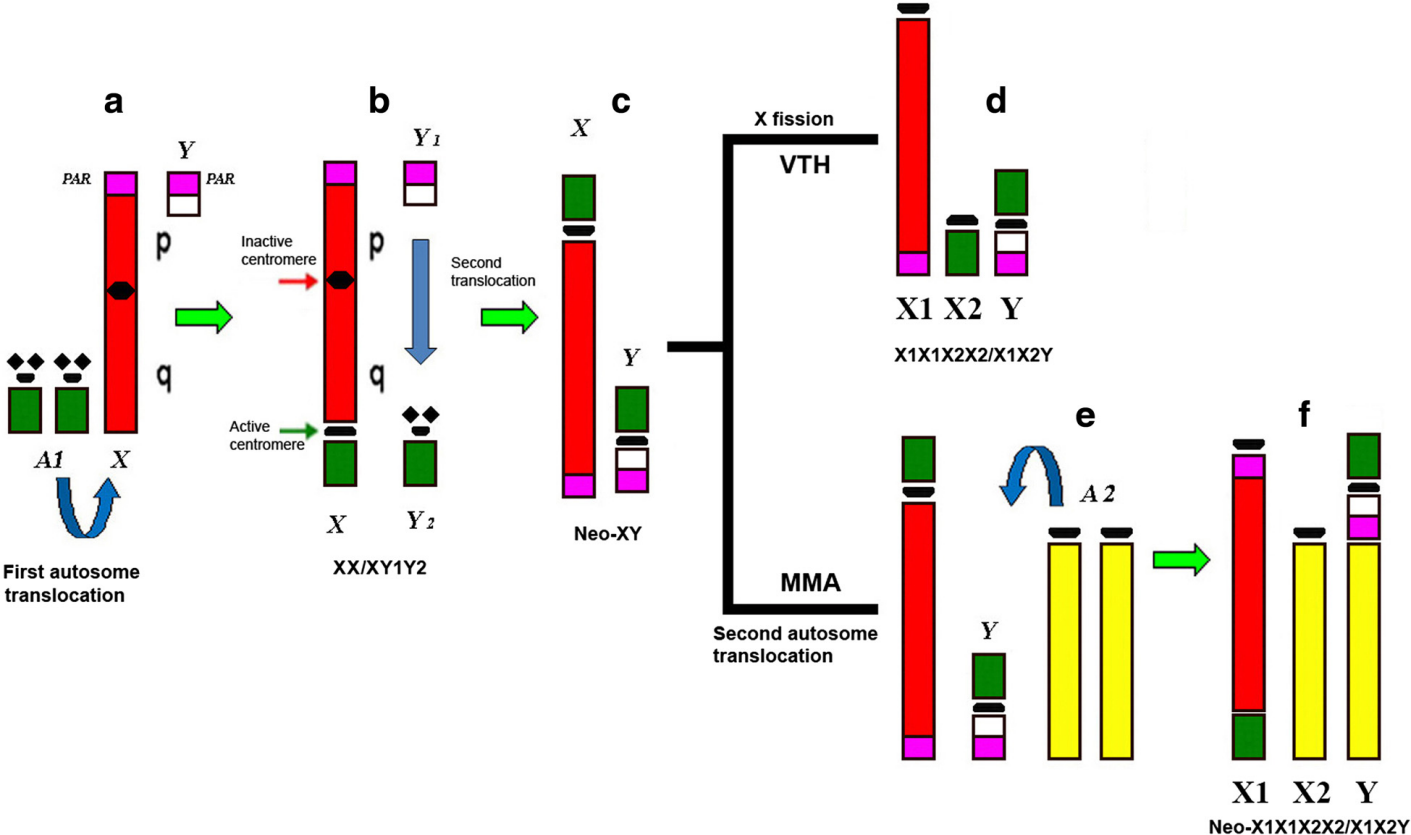
Sex chromosome evolution in the subfamily Stenodermatinae. **a** The original XY system found in most mammals. **b** Fusion of one autosome with X, originating the XX/XY1Y2 system found, for instance, in AOB. **c** Neo-XY system after the fusion of the free homologue (called Y2) with Y (called Y1), found in UMA and UBI and probably the common ancestor of *Vampyressa* and *Mesophylla*. **d** Fission at centromere of Neo-X, freeing the autosome portion once more, originating the X1X1X2X2/X1X2Y system in VTH. **e** Fusion of a second autosome to Y of Neo-XY. **f** The resulting Neo- X1X1X2X2/X1X2Y in MMA

We suggest that all species in the Vampyressina have a Neo-XY sex chromosome system and that the different sex chromosome systems as seen in *Mesophylla* and *Vampyressa* represent a derived character state (Fig. 3). In the genus *Mesophylla,* the sex chromosome system has evolved from a Neo-XY system after a further translocation of the compound Y with an autosome that led to a Neo-X1X2Y. To our knowledge, it has never been described before in mammals. In *V. thyone,* the sex chromosomes system evolved after the fission of the autosome (corresponding to PHA15) that was translocated to the X chromosome in the ancestral karyotype of Stenodermatinae. As the Y-autosome translocation also involves an autosome, its free homologue would be X2.

## Conclusions

Our comparative analysis of all genera of vampyressine using a cytogenomic approach confirmed the molecular phylogeny previously described for this group, being independent evidence supporting that phylogeny. Our results also confirm that all genera of this subtribe have compound sex chromosome systems that originated from an X-autosome translocation, an ancestral condition observed in the Stenodermatinae subfamily. In *Mesophylla* we found a sex chromosome system never described before in mammals. We were able to trace all the chromosomal sex system evolution in vampyressine and show that it mirrors the phylogeny of this group. This work presents additional data supporting Hoofer & Baker (2006 [9]) hypothesis based on molecular studies regarding the polyphyly of the genus *Vampyressa* and its sister relationship to *Mesophylla*.

## Abbreviations

2n, diploid number; Ag-NOR, silver nitrate staining; AOB, *Artibeus obscurus*; CBR, *Carollia brevicauda;* CH, constitutive heterochromatin; CVI, *Chiroderma villosum*; DAPI, 4’, 6-diamidino-2-phenylindole; DEC, *Diphylla ecaudata*; DRO, *Desmodus rotundus*; DOP-PCR, Degenerate oligonucleotide-primed-polymerase chain reaction; DYO, *Diaemus youngi*; FISH, *Fluorescence In Situ Hybridization*; FN, Fundamental Number; MP, Maximum parsimony ; NOR, Nucleolar organizer region; PHA, *Phyllostomus hastatus;* PIN, *Platyrrhinus incarum*; MMA, *Mesophylla macconnelli;* PAUP, Phylogenetic analysis using parsimony; SSC, Saline sodium citrate; TBR, tree bisection reconnection; TNT, Tree analysis using new technology; VBI, *Vampyriscus bidens*; VBR, *Vampyriscus brocki;* VCA, *Vampyrodes caraccioli*; VTH, *Vampyressa thyone.*

## Additional files

Additional file 1: Table S1. Vampyressina, Basic Data Matrix using PHA chromosome numbering as reference (DOC 198 kb) Additional file 2: Figure S1. C-banding patterns on *Chiroderma villosum* (CVI); *Vampyriscus bidens* (VBI); *Vampyressa thyone* (VTH); *Mesophylla macconnelli* (MMA); *Vampyrodes caraccioli* (VCA); *Platyrrhinus incarum* (PIN); *Vampyriscus brocki* (VBR). (JPG 1943 kb) Additional file 3: Figure S2. Fish with telomeric (left) and rDNA probes (right) in *Chiroderma villosum* (CVI); *Vampyriscus bidens* (VBI); *Vampyriscus brocki* (VBR); *Vampyressa thyone* (VTH); *Mesophylla macconnelli* (MMA); *Platyrrhinus incarum* (PIN); *Vampyrodes caraccioli* (VCA). (JPG 5731 kb) Additional file 4: Figure S3. G-banding patterns of *Platyrrhinus incarum* (PIN) and *Vampyrodes caraccioli* (VCA). (JPG 807 kb)
