# Supplementary figures and images for: Chromosomal phylogeny of Vampyressine bats (Chiroptera, Phyllostomidae) with description of two new sex chromosome systems
Source: BMC Evol Biol. 2016 Jun 4;16:119. doi: 10.1186/s12862-016-0689-x (PMC4893233; doi:10.1186/s12862-016-0689-x)

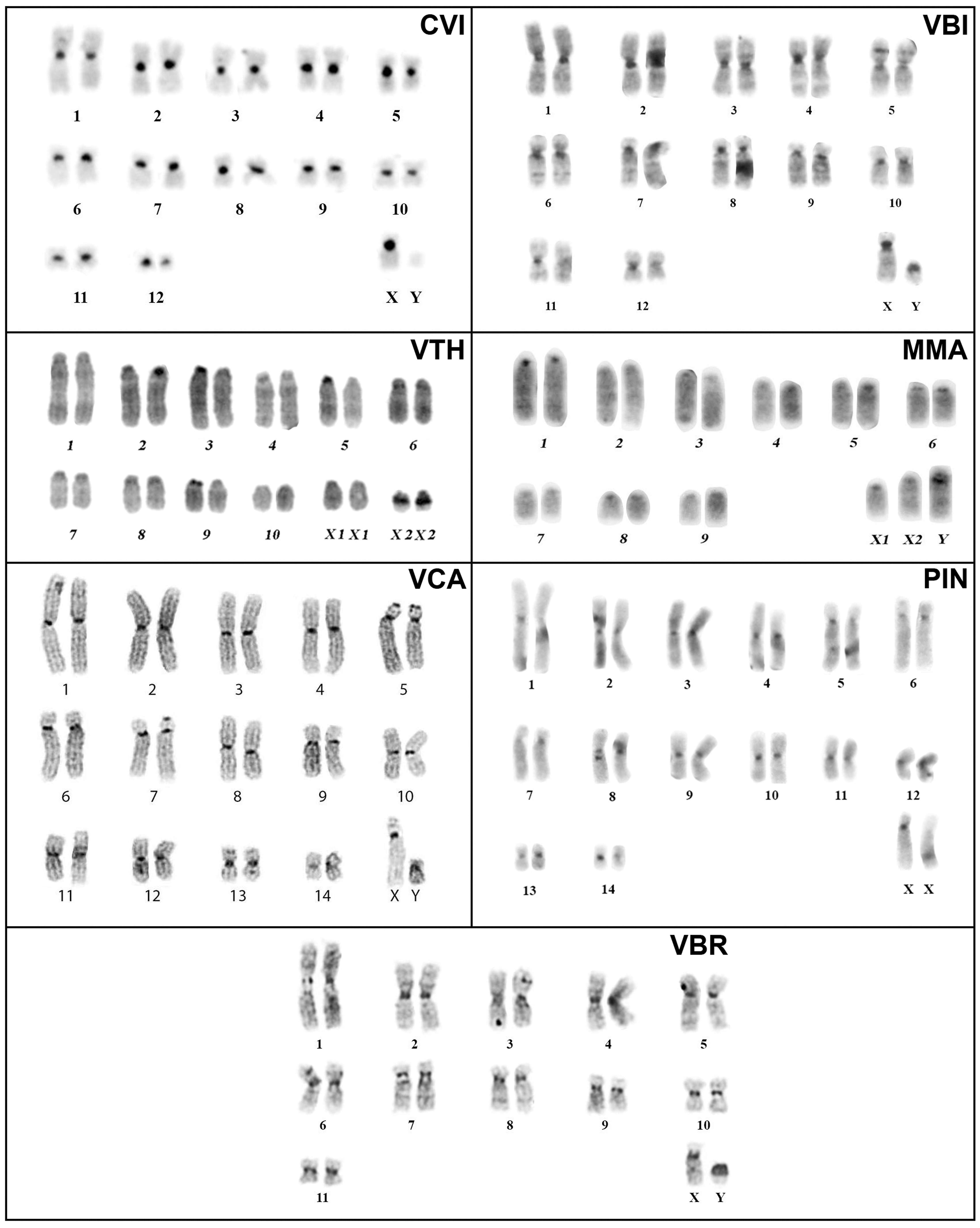

Supplement: Additional file 2: Figure S1. — C-banding patterns on Chiroderma villosum (CVI); Vampyriscus bidens (VBI); Vampyressa thyone (VTH); Mesophylla macconnelli (MMA); Vampyrodes caraccioli (VCA); Platyrrhinus incarum (PIN); Vampyriscus brocki (VBR). (JPG 1943 kb) [file 12862_2016_689_MOESM2_ESM.jpg]

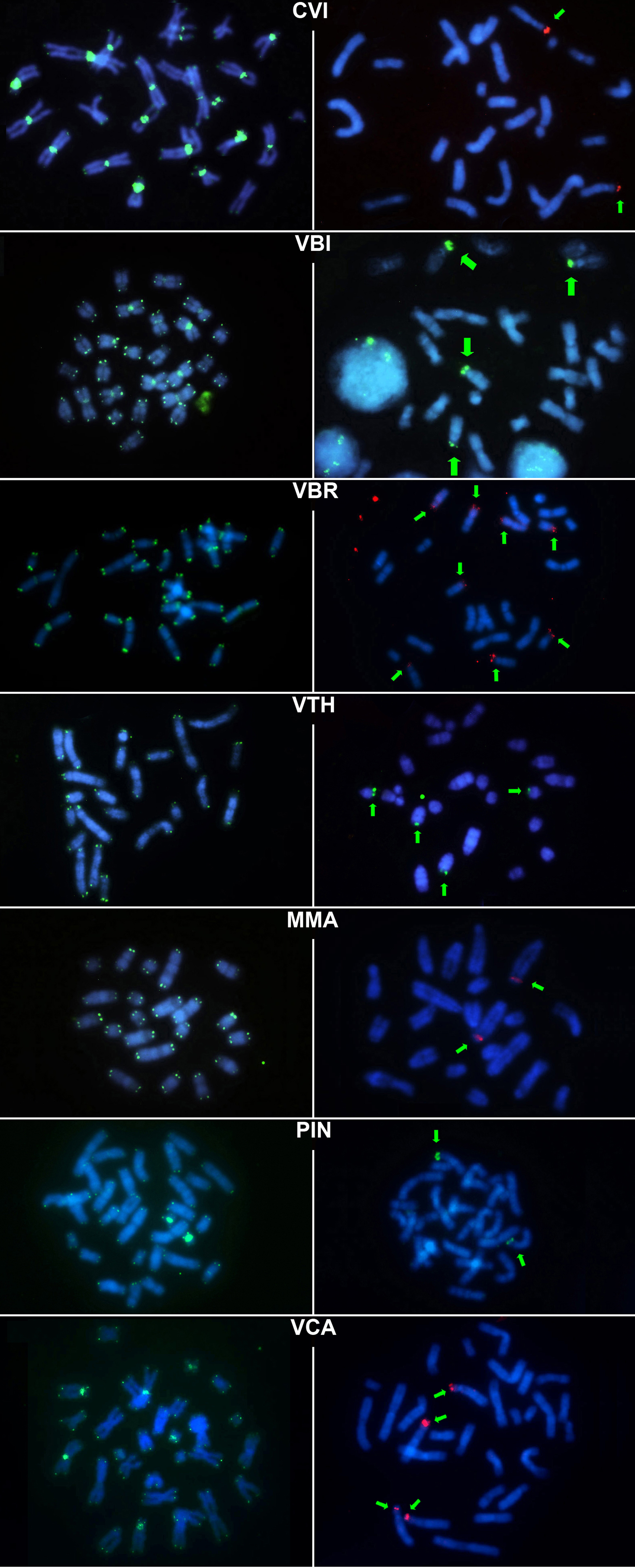

Supplement: Additional file 3: Figure S2. — Fish with telomeric (left) and rDNA probes (right) in Chiroderma villosum (CVI); Vampyriscus bidens (VBI); Vampyriscus brocki (VBR); Vampyressa thyone (VTH); Mesophylla macconnelli (MMA); Platyrrhinus incarum (PIN); Vampyrodes caraccioli (VCA). (JPG 5731 kb) [file 12862_2016_689_MOESM3_ESM.jpg]

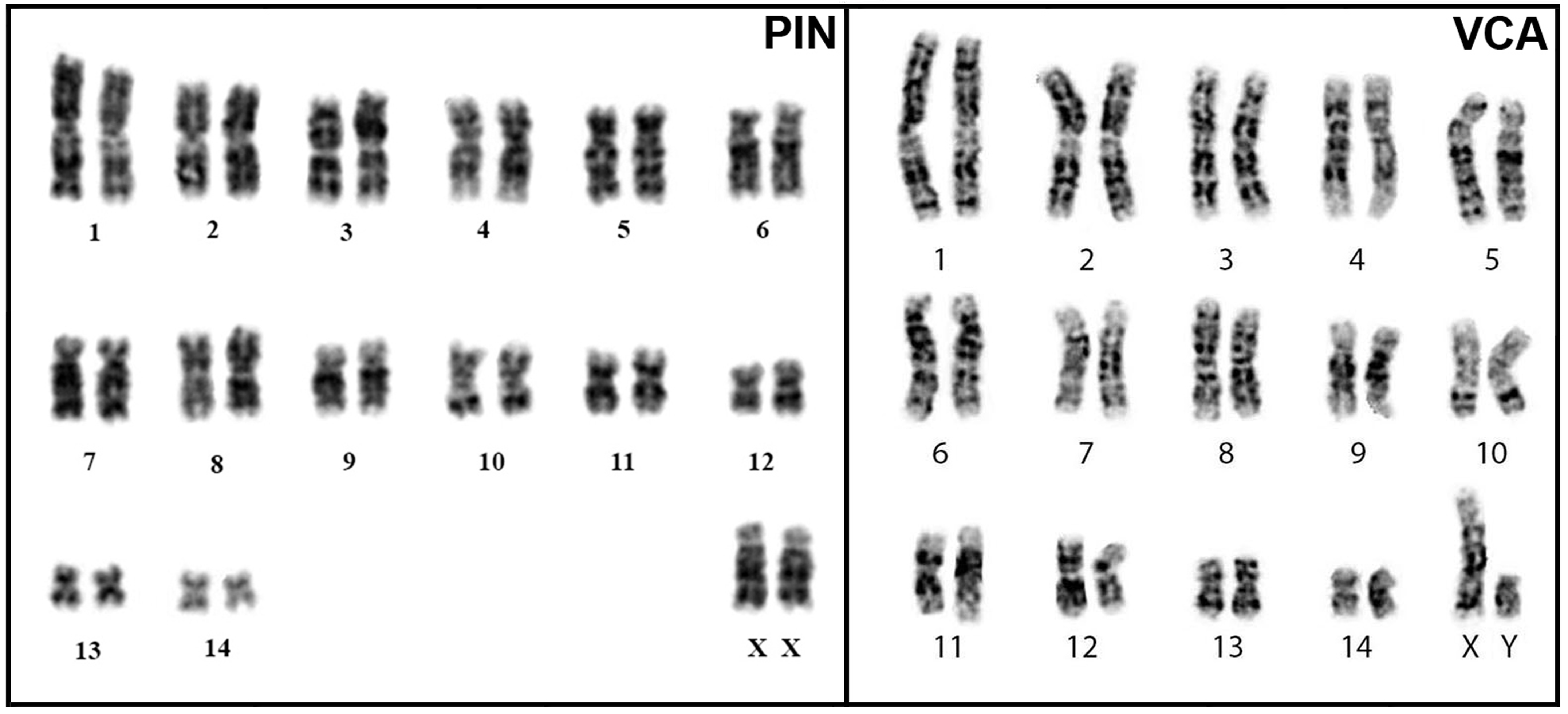

Supplement: Additional file 4: Figure S3. — G-banding patterns of Platyrrhinus incarum (PIN) and Vampyrodes caraccioli (VCA). (JPG 807 kb) [file 12862_2016_689_MOESM4_ESM.jpg]
